# Mouse Model of Loeys–Dietz Syndrome Shows Elevated Susceptibility to Periodontitis via Alterations in Transforming Growth Factor-Beta Signaling

**DOI:** 10.3389/fphys.2021.715687

**Published:** 2021-08-11

**Authors:** Satoru Yamada, Kenichiro Tsushima, Masaki Kinoshita, Hiromi Sakashita, Tetsuhiro Kajikawa, Chiharu Fujihara, Hang Yuan, Shigeki Suzuki, Takayuki Morisaki, Shinya Murakami

**Affiliations:** ^1^Department of Periodontology, Osaka University Graduate School of Dentistry, Suita, Japan; ^2^Department of Periodontology and Endodontology, Tohoku University Graduate School of Dentistry, Sendai, Japan; ^3^Division of Molecular Pathology, Laboratory of Genome Technology IMSUT Hospital, Department of Internal Medicine, Human Genome Center, The Institute of Medical Science, The University of Tokyo, Bunkyo, Japan

**Keywords:** TGF-beta, periodontal ligament, extracelluar matix, periodontitis, knock-in mice

## Abstract

Loeys–Dietz syndrome (LDS) is a syndromic connective tissue disorder caused by a heterozygous missense mutation in genes that encode transforming growth factor (TGF)-β receptor (*TGFBR*) *1* and *2*. We encountered a patient with LDS, who had severe periodontal tissue destruction indicative of aggressive periodontitis. The patient had a missense mutation in the glycine and serine-rich domain of *TGFBR1* exon 3. This G-to-T mutation at base 563 converted glycine to valine. We established an LDS model knock-in mouse that recapitulated the LDS phenotype. Homozygosity of the mutation caused embryonic lethality and heterozygous knock-in mice showed distorted and ruptured elastic fibers in the aorta at 24 weeks of age and died earlier than wildtype (WT) mice. We stimulated mouse embryonic fibroblasts (MEFs) from the knock-in mouse with TGF-β and examined their responses. The knock-in MEFs showed downregulated *Serpine 1* mRNA expression and phosphorylation of Smad2 to TGF-β compared with WT MEFs. To clarify the influence of TGF-β signaling abnormalities on the pathogenesis or progression of periodontitis, we performed pathomolecular analysis of the knock-in mouse. There were no structural differences in periodontal tissues between WT and LDS model mice at 6 or 24 weeks of age. Micro-computed tomography revealed no significant difference in alveolar bone resorption between WT and knock-in mice at 6 or 24 weeks of age. However, TGF-β-related gene expression was increased significantly in periodontal tissues of the knock-in mouse compared with WT mice. Next, we assessed a mouse periodontitis model in which periodontal bone loss was induced by oral inoculation with the bacterial strain *Porphyromonas gingivalis* W83. After inoculation, we collected alveolar bone and carried out morphometric analysis. *P. gingivalis*-induced alveolar bone loss was significantly greater in LDS model mice than in WT mice. Peritoneal macrophages isolated from *Tgfbr1^*G*188V/+^* mice showed upregulation of inflammatory cytokine mRNA expression induced by *P. gingivalis* lipopolysaccharide compared with WT macrophages. In this study, we established an LDS mouse model and demonstrated that LDS model mice had elevated susceptibility to *P. gingivalis*-induced periodontitis, probably through TGF-β signal dysfunction. This suggests that TGF-β signaling abnormalities accelerate the pathogenesis or progression of periodontitis.

## Introduction

Marfan syndrome (MFS) is an autosomal dominant connective tissue disease caused by a mutation in fibrillin-1 (Dietz et al., 1991), which leads to systemic diseases with various phenotypes. It affects approximately 1 in 5,000 people and includes patients with mild disease (Dietz et al., 1991). In particular, MFS causes characteristic signs in the skeletal system (e.g., bone hyperplasia, joint relaxation, height, arachnoid finger, and spinal scoliosis), cardiovascular system (e.g., aortic aneurysm and mitral valve deviation), and ocular system (e.g., lens subluxation) (Schorr et al., 1951). Fibrillin-1 is a component of microfibrils in the extracellular matrix (Godfrey et al., 1990) and is involved in the control of transforming growth factor (TGF)-β expression and function (Neptune et al., 2003). TGF-β is a cytokine involved in the regulation of cell proliferation, differentiation, and death (Huang and Chen, 2012). In particular, it promotes collagen production and extracellular matrix remodeling (Dietz, 2007). Various MFS symptoms result from excessive TGF-β in serum caused by fibrillin-1 mutations (Matt et al., 2009). Additionally, various MFS-related diseases are caused by abnormalities in TGF-β signaling, which include Beals syndrome caused by mutations in *fibrillin-2* (Putnam et al., 1995), Loeys–Dietz syndrome (LDS) caused by mutations in *TGF-*β *receptor* (*TGFBR*) *1* or *TGFBR2* (Mizuguchi et al., 2004; Loeys et al., 2005, 2006), juvenile polyposis syndrome caused by mutations in *Smad4*, a TGF-β signaling factor (Howe et al., 1998), and Shprintzen–Goldberg syndrome caused by mutations in *SKI*, a gene that suppresses Smad signaling (Doyle et al., 2012).

There have been reports of patients with MFS and severe periodontitis (De Coster et al., 2002; Straub et al., 2002; Jain and Pandey, 2013; Staufenbiel et al., 2013). Patients with MFS have greater morbidity and severity of periodontitis than otherwise healthy individuals (Suzuki et al., 2015). In patients who exhibit MFS, severe chronic periodontitis has been reported with periodontitis substantial palatal and dental irregularities. To our knowledge, details of the relationships between genetic mutations and periodontitis in MFS patients and its related diseases are unclear. Thus, in this study, we first investigated the relationships between MFS, related diseases, and periodontitis. We identified a patient with LDS, who had an aggressive periodontitis-like pathology. Periodontitis progresses under a range of conditions that include environmental and genetic factors. Periodontitis is broadly divided into chronic and aggressive periodontitis, with aggressive periodontitis assumed to be more closely related to genetic factors (Meng et al., 2007). Recently, a new periodontitis classification scheme has been adopted, in which forms of the disease recognized as “chronic” or “aggressive” are now characterized by a multi-dimensional staging and grading system (Papapanou et al., 2018).

LDS is caused by mutations in *TGFBR1* or *TGFBR2* (Loeys et al., 2005). LDS and MFS share many common clinical symptoms that include aortic lesions (e.g., basal dilatation, aortic aneurysm, and aortic dissection) and skeletal system lesions (e.g., scoliosis, joint laxity, and spider finger). However, features not evident in patients with MFS (i.e., characteristic facial features such as cleft palate, dichotomy uvula, interocular dissociation, systemic blood vessel meandering, craniosynostosis, congenital heart disease, and intellectual disability) are observed at high rates in patients with LDS (Loeys et al., 2006). In vascular smooth muscle cells collected from patients with LDS, TGF-β levels are low (Gallo et al., 2014). However, a compensatory change comprises TGF-β overexpression in aortic tissues. This likely results in the onset of cardiovascular symptoms such as aortic aneurysms and aortic dissection (Loeys et al., 2005; Gallo et al., 2014). On the basis of the data collected from the LDS patient who had aggressive periodontitis, we developed genetically modified mice with the candidate genetic mutation to analyze the disease state and the effects of a mutation in a TGF-β-related gene on periodontitis.

## Materials and Methods

### Patients and Mutation Analysis

All human experiments were approved by the Institutional Ethics Committee of Osaka University Graduate School of Dentistry (No. H22-E10) and the National Cerebral and Cardiovascular Center (No. M22-34). The epidemiological study included 120 patients who had visited the National Cerebral and Cardiovascular Center Hospital in Osaka, Japan, and were diagnosed with Marfan syndrome or Marfan-related syndrome by the revised Ghent nosology (Loeys et al., 2010) to survey the prevalence rate of periodontal disease in Marfan syndrome and Marfan-related syndrome patients. Informed consent was obtained from all patients involved in the study. Genomic DNA isolated from peripheral white blood cells was amplified by polymerase chain reaction (PCR) using primers in the flanking introns of *TGFBR1* and *TGFBR2*. Sequence analyses were performed using Applied Biosystems automated DNA sequencer (ABI3770, Waltham, MA, United States) in accordance with the manufacturer’s protocol.

### Animals

All animal experiments were approved by the Institutional Animal Care and Use Committee of Osaka University Graduate School of Dentistry and complied with the guidelines for the care and use of laboratory animals at Osaka University. This study was carried out in compliance with the ARRIVE guidelines, where applicable. To generate *Tgfbr1^*G*188V/+^* mice, site-directed mutagenesis was performed to replace glycine with valine at codon 188 of *Tgfbr1* (guanine to thymine at nucleotide 563). This mutated *Tgfbr1* cDNA was cloned into pBSIISK + with a floxed neomycin resistance cassette (NeoR). Bac-based long-range PCR was used to amplify murine genomic fragments of *Tgfbr1*. The long arm (6 kb, exon 3 with G188V) and short arm (2.8 kb, exon 4) were cloned into pBS-DTA and pBS-LNL, respectively. The final targeting vector was constructed and then linearized. The targeting vector DNA was electroporated into C57BL/6 ES cells. Homologous recombination-positive ES cells were identified by Southern blot analysis. Positive clones were injected into BALB/c blastocysts and transferred into pseudopregnant female mice. Chimeric offspring were mated with C57BL/6 mice and germline transmission was confirmed by RT-PCR. The loxP-flanked NeoR was removed by mating *Tgfbr1^*G*188V/+^* founder mice with CAG-Cre recombinase transgenic mice (Sakai and Miyazaki, 1997). Experimental mice were backcrossed with C57BL/6 mice to remove the CAG-Cre transgene. Genotype analysis of *Tgfbr1^*G*188V/+^* mice was performed by genomic PCR using flanking loxP site-specific primers (5′CTAAGAGAAGTGTGCCTCCTTTACA-3′ and 5′-CCAAAGTCATAGAGCATGTGTTAGA-3′).

### Cell Culture and Gene Transfection

Wildtype (WT) and *Tgfbr1^*G*188V/+^* mouse embryonic fibroblasts (MEFs) were isolated from embryos on day 13.5 by a previously described method (Awata et al., 2015). MEFs were cultured in Dulbecco’s modified Eagle’s medium (DMEM) supplemented with 10% fetal bovine serum (FBS) and 60 μg/mL kanamycin. MEFs from passages 3–5 were used in this study. Each genotype of MEFs (WT, *Tgfbr1^*G*188V/+^*, and *Tgfbr1^*G*188V/G188V^*) was cultured in a 12-well plate until confluency. The next day, the medium was replaced with serum-free DMEM. After serum deprivation for 24 h, the cells were stimulated with TGF-β (R&D Systems, Minneapolis, MN, United States) in serum-free DMEM for 30 min for western blot analysis and 12 h for quantitative PCR analysis.

cDNA of the mouse *Tgfbr1* gene was cloned into the p3XFLAG-CMV-14 expression vector (Sigma-Aldrich, St. Louis, MO, United States). cDNA for the *Tgfbr1* mutation (G188V: *Tgfbr1^*G*188V^*) was ligated to *Tgfbr1* cDNA by the Quick Change Site-Directed Mutagenesis kit (Stratagene, LA Jolla, CA, United States) in accordance with the manufacturer’s protocol. The sequence was verified by DNA sequencing. For luciferase assays, human embryonic kidney (HEK) 293 cells were seeded in a 12-well plate. After 24 h, the cells were transfected with the *Tgfbr1^*G*188V^*/3XFLAG-CMV-14 expression vector or wildtype *Tgfbr1*/3XFLAG-CMV-14 expression vector mixed with Transcription Factor Reporter using a Signal SMAD Reporter Assay kit (Polyscience, Inc., Warrington, PA, United States) in accordance with the manufacturer’s protocol. At 48 h after transfection, the medium was replaced with serum-free DMEM. After serum deprivation for 24 h, the cells were stimulated with 0–5 ng/ml TGF-β in serum-free DMEM for 8 h. Luciferase activity was measured by a GloMax 96 Microplate Luminometer (Promega, Madison, WI, United States).

### Histological Analysis

Thoracic aortae were collected from WT and *Tgfbr1^*G*188V/+^* mice at 24 weeks of age and fixed overnight in 4% paraformaldehyde (PFA)/phosphate buffer (Wako Pure Chemical Industries, Osaka, Japan). Samples were embedded in paraffin and sectioned at 5 μm thicknesses using a LEICA RM2245 microtome (Leica Microsystems, Wetzlar, Germany). Sections were stained with Elastica van Gieson (EVG) for elastin staining. Maxillae from WT and *Tgfbr1^*G*188V/+^* mice at 6 and 24 weeks of age were fixed in 4% PFA/phosphate-buffered saline (PBS) (Wako Pure Chemical Industries) overnight at 4°C and decalcified in 0.5 M EDTA (Wako) for 1 week. After decalcification, periodontal tissues were dehydrated using 15, 20, and 25% sucrose in PBS. Then, periodontal tissues were embedded in O.C.T. Compound (Sakura Finetek, Tokyo, Japan). They were frozen and sectioned at 5 μm thicknesses in a mesiodistal orientation using the LEICA RM2245 microtome. Sections were stained with hematoxylin-eosin (HE) or using a Tartrate-Resistance Acid Phosphatase (TRAP) Staining Kit (Wako) in accordance with the manufacturer’s protocol.

### RNA Extraction and Quantitative PCR Analysis

Thoracic aortae, maxillae, and periodontal tissues were extracted from 6-week-old male WT and *Tgfbr1^*G*188V/+^* mice. Total RNA from tissues or MEFs was extracted using a PureLink RNA Mini Kit (Life Technologies, Carlsbad, CA, United States). Total RNA was reversed transcribed to cDNA using a High-Capacity RNA-to-cDNA Kit (Applied Biosystems, Foster City, CA, United States). Quantitative PCR was performed with the StepOnePlus Real-time PCR system (Applied Biosystems) using Fast SYBR Green Master Mix (Thermo Fisher Scientific, Waltham, MA, United States) and gene-specific primers (Table 1).

**TABLE 1:** Primer sequences.

**GenBank acc.**	**Gene**	**Primer sequences**
NM_009370	*Tgfb1*	5′-GATGTCAGCTCTGGGCAAAGATTAG-3′ 5′-CAGGCTGAGCTTCATGCCTTTAC-3′
NM_009371	*Tgfb2*	5′-AATGGTTGCACCACAAGCAAGA-3 5′-TTCCCAGGGCTGAGATGATAAGAG-3′
NM_009368	*Tgfb3*	5′-CAGCGCTACATAGGTGGCAAGA-3′ 5′-TGATTTCCAGACCCAAGTTGGAC-3′
NM_009370	*Tgfbr1*	5′-GATGTCAGCTCTGGGCAAAGATTAG-3′ 5′-CAGGCTGAGCTTCATGCCTTTAC-3′
NM_009371	*Tgfbr2*	5′-AGTCGGATGTGGAAATGGAA-3′ 5′-ACAGCTGTGGAAGCTTGACC- 3′
NM_007742	*Col1a1*	5′-CAGGGTATTGCTGGACAACGTG-3′ 5′-GGACCTTGTTTGCCAGGTTCA-3′
NM_008871	*Serpine1*	5′-TGCTGAACTCATCAGACAATGGAAG-3′ 5′-TCGGCCAGGGTTGCACTAA-3′
NM_025711	*Plap-1*	5′-CCATATCAGGATCGCTGAAGCA-3′ 5′-TCTGTGATTCTGTTGTTTCCAAGAC-3′
NM_021274	*Cxcl10*	5′-TGAATCCGGAATCTAAGACCATCAA-3′ 5′-AGGACTAGCCATCCACTGGGTAAAG-3
NM_031168	*Il6*	5′- CCACTTCACAAGTCGGAGGCTTA-3′ 5′-GCAAGTGCATCATCGTTGTTCATAC-3′
NM_013693	*Tnf*	5′-CAGGAGGGAGAACAGAAACTCCA-3′ 5′-CCTGGTTGGCTGCTTGCTT-3′

### Western Blot Analysis

Cells were lysed with RIPA buffer (Millipore, Billerica, MA, United States) that contained phosphatase and proteinase inhibitors. The protein concentration of the lysates was measured by the Bradford assay (Bio-Rad, Hercules, CA, United States). Aliquots of lysates were separated by 10% sodium dodecyl sulfate-polyacrylamide gel electrophoresis and subjected to western blot analysis. Primary antibodies included a rabbit anti-TGFBR1 antibody (1:1,000; Santa Cruz Biotechnology, Santa Cruz, CA, United States), mouse anti-beta actin antibody (1:10,000, Sigma-Aldrich), rabbit anti-phospho-Smad2 antibody (1:1,000, Millipore), and rabbit anti-Smad2 antibody (1:1,000, Cell Signaling Technology, Danvers, MA, United States). Secondary antibodies were a horseradish peroxidase (HRP)-linked anti-mouse IgG antibody (1:10,000, GE Healthcare, Piscataway, NJ, United States) and HRP-linked anti-rabbit IgG antibody (1:10,000, GE Healthcare). Immunoreactive proteins were detected by SuperSignal West Pico Chemiluminescent Substrate (Thermo Fisher Scientific, Waltham, MA, United States) with an ImageQuant LAS4,000 (GE Healthcare).

### Quantitative Analysis of Alveolar Bone Resorption

Maxillae were collected from WT and *Tgfbr1^*G*188V/+^* mice at 6 and 24 weeks of age and imaged by a 3D micro X-ray CT R_mCT2 (Rigaku, Tokyo, Japan). The images were analyzed using TRI/3D-BON software (RATOC System Engineering, Tokyo, Japan). The root surface area between the alveolar bone crest and cementoenamel junction was measured using WinROOF software (Mitani, Fukui, Japan). The total value of these three distances was regarded as alveolar bone resorption. Alveolar bone resorption was measured in the root surface area between the alveolar apex from the cementoenamel junction.

### *Porphyromonas gingivalis* Bacterial Culture and Establishment of Oral Infection

*Porphyromonas gingivalis* strain W83 was cultured in modified Gifu anaerobic medium broth (Nissui, Tokyo, Japan) in an anaerobic jar (Becton Dickinson Microbiology System, Cockeysville, MD, United States) in the presence of an AnaeroPack (Mitsubishi Gas Chemical Co., Inc., Tokyo, Japan) for 48 h at 37°C. Bacterial suspensions were prepared in PBS without Mg^2+^/Ca^2+^ using established growth curves and spectrophotometric analysis. The number of CFUs was standardized by measuring optical density at 600 nm. The murine experimental periodontal infection model was established in accordance with a previously described method (Aoki-Nonaka et al., 2013). The 8-week-old male mice received sulfamethoxazole and trimethoprim at final concentrations of 700 and 400 μg/mL, respectively, in water bottles administered *ad libitum* for 10 days. This treatment was followed by 3 days without antibiotics. The experimental group was then infected as follows. In total, 1 × 10^9^ CFUs of live *P. gingivalis* were suspended in 100 μL PBS with 2% carboxymethyl cellulose (Sigma−Aldrich) and administered to each mouse through a feeding needle 10 times at 3-day intervals. The control group received the same pretreatment and was sham infected without *P. gingivalis*. At 1 day after the final treatment, the mice were sacrificed with CO_2_ affixation and their maxillae were collected for micro-CT and histological analyses.

### *P. gingivalis* Stimulation of Peritoneal Macrophages Derived From *Tgfbr1^*G*188V/+^* Mice

Macrophages were harvested from the peritoneal cavity of 8-week-old male WT and *Tgfbr1^*G*188V/+^* mice at 3 days after injection of thioglycollate (Yamaba et al., 2015) and seeded on 6-well plates. After 2 h of incubation, non-adherent cells were washed out and the remaining cells were subjected to further analysis. The peritoneal macrophages were stimulated with 1 Uμg/ml *P. gingivalis* lipopolysaccharide (LPS) (InvivoGen, Inc., San Diego, CA, United States) for the indicated times and then total RNA was isolated for quantitative PCR analysis.

### Statistical Analysis

Data are represented as the mean ± SD. Statistical analyses were performed using the Student’s *t*-test for paired comparisons and one-way analysis of variance for multiple comparisons using Bonferroni’s *post hoc* test with Excel statistics software (Bellcurve, Tokyo, Japan). A value of *P* < 0.05 was considered statistically significant.

## Results

### Mutation Analysis of the LDS Patient With Periodontitis

**FIGURE 1:**
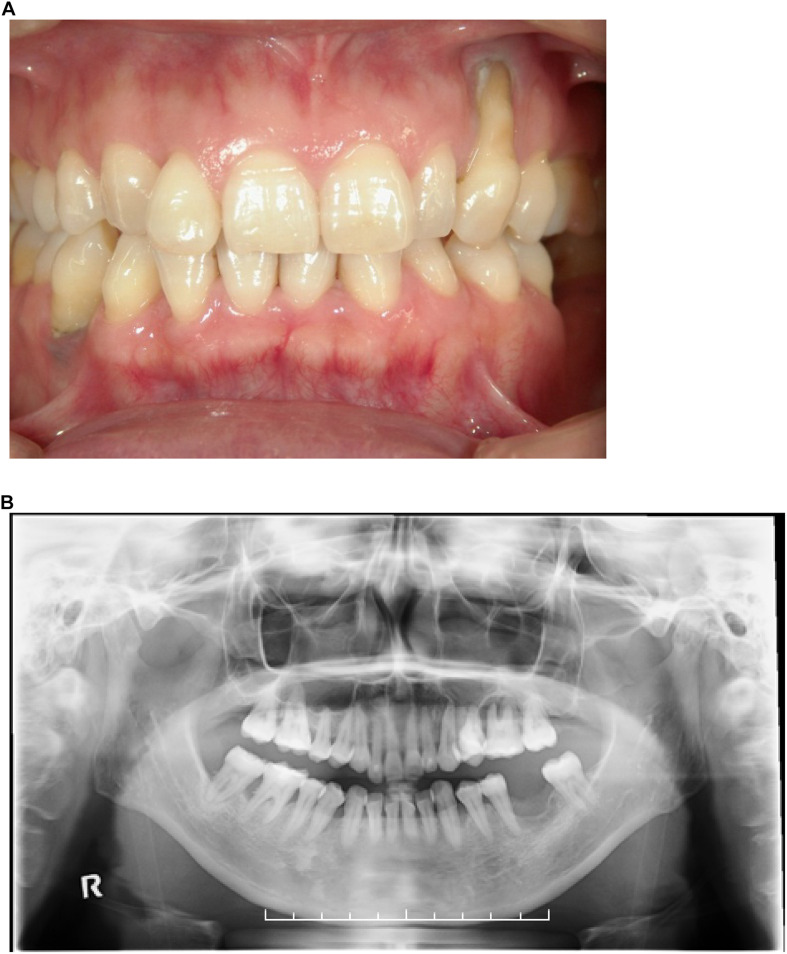
**(A)** Front view of the dentition of a patient with LDS and aggressive periodontitis. **(B)** Orthopantomograph of the patient. Note the presence of localized vertical bone loss around molars.

**FIGURE 2:**
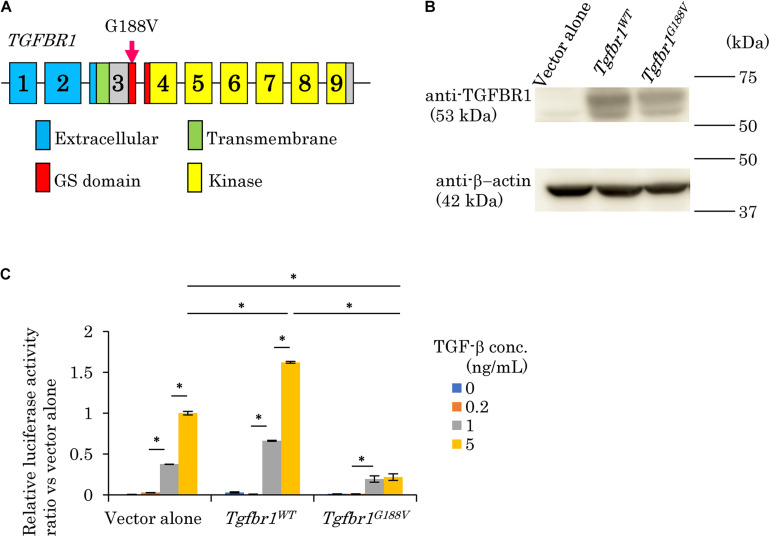
Mutation in exon 3 of *TGFBR1* isolated from a patient with LDS and aggressive periodontitis diminishes the response to TGF-β (G188V mutation). **(A)** Location of the mutation in exon 3 of *TGFBR1*. **(B)** HEK293 cells were transfected with a vector alone, wildtype mouse *Tgfbr1* (*Tgfbr1*^*WT*^), or mouse *Tgfbr1* with the G188V mutation (*Tgfbr1^*G*188V^*). Lysates of transfected cells were subjected to western blot analysis using an anti-TGFBR1 antibody. **(C)** TGF-β-induced promoter activity was assessed by the dual luciferase reporter assay system. Values represent the mean ± SD in triplicate assays. ^∗^*P* < 0.05, compared with the indicated TGF-β concentration.

We surveyed 120 patients with Marfan syndrome or Marfan-related syndrome to determine the prevalence rate of periodontal disease in these syndromes. We found that the prevalence of chronic periodontitis in Marfan syndrome and Marfan-related syndrome patients was significantly higher compared with that in healthy controls (manuscript in preparation). In this clinical study, we encountered a 44-year-old Japanese female with LDS, who had localized vertical bone loss around molars in spite of good oral hygiene (O’Leary’s plaque control record: 13.0%) as shown in Figure 1. She was only one patient diagnosed with aggressive periodontitis (new classification: stage III and grade B). She was diagnosed with familial thoracic aortic aneurysms and dissections. She was treated by total arch aortic and descending aorta replacement. Systemic features of the patient were a tall height (−), down slanting palpebral fissures (+), retrognathia (+), pectus carinatum deformity (−), pneumothorax (−), myopia (−), hypertelorism (+), and bifid uvula (−). Genomic sequencing of the patient revealed a mutation in the glycine and serine-rich domain of *TGFBR1* exon 3 (referred to as the GS domain). This G-to-T mutation at base 563 converted glycine to valine at residue 188 (G188V) (Figure 2A). To assess the effects of the G188V mutation on TGF-β signaling, we introduced mouse *Tgfbr1^*WT*^* and *Tgfbr1^*G*188V^* expression vectors into HEK293 cells. We confirmed that the TGF-β type I receptor was sufficiently expressed in both groups (Figure 2B). Next, we performed luciferase assays and found that TGF-β-induced luciferase activity was significantly elevated in the group with high levels of *Tgfbr1^*WT*^* and significantly suppressed in the group with *Tgfbr1^*G*188V^* (Figure 2C). These results suggested that the G188V mutation resulted in loss of TGF-β signaling.

### Generation of *Tgfbr1^*G*188V^* Knock-In Mice

**FIGURE 3:**
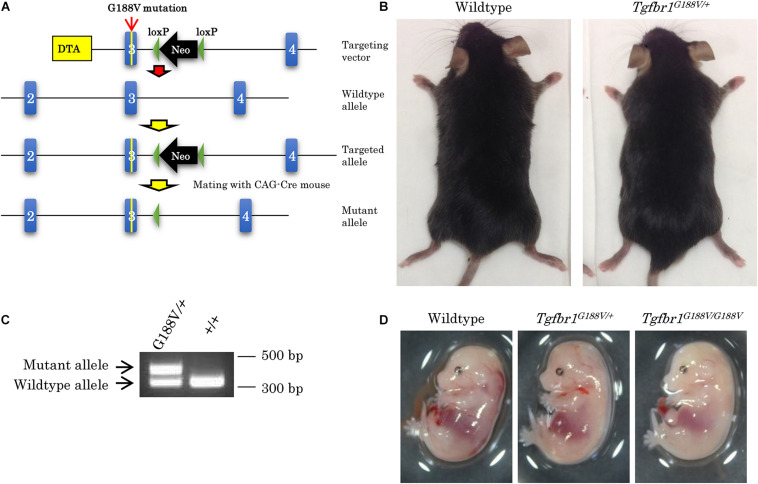
Generation of knock-in mice with the *Tgfbr1* G188V mutation. **(A)** Structure of the *Tgfbr1* G188V mutant allele. **(B)** No clear differences in 6-week-old *Tgfbr1*^*G*188V/+^ mice. **(C)** PCR genotyping of genomic DNA using tail specimens collected from *Tgfbr1^*G*188V/+^* and WT mice. **(D)** Embryos of WT, *Tgfbr1^*G*188V/+^*, and *Tgfbr1^*G*188V/G188V^* at embryonic day 14.5.

To introduce the G188V mutation into mice, we generated a knock-in construct (Figure 3A). *Tgfbr1^*G*188V/+^* mice were delivered spontaneously, able to breed, and were fertile with no apparent abnormalities at 6 weeks of age (Figure 3B). Mouse genotyping was performed using genomic DNA by PCR with a primer to identify the *Tgfbr1* allele and mutant *Tgfbr1* allele that contained the loxP sequence (Figure 3C). After generation of *Tgfbr1^*G*188V/+^* mice, genotyping was performed using embryos on days 13.5 and 14.5, and after birth. WT, *Tgfbr1^*G*188V/+^*, and *Tgfbr1^*G*188V/G188V^* embryos were obtained at day 14.5 (Figure 3D). We observed no conspicuous morphological or patterning defects, although some *Tgfbr1^*G*188V/G188V^* embryos at day 14.5 showed fewer bloodstreams in craniofacial, trunk, and spinal cord regions. Among embryos on days 13.5 and 14.5, WT, *Tgfbr1^*G*188V/+^*, and *Tgfbr1^*G*188V/G188V^* mice had survived. However, with respect to the number of births, only one *Tgfbr1^*G*188V/G188V^* mouse was obtained. Moreover, the *Tgfbr1^*G*188V/G188V^* mutation caused embryonic lethality after day 14.5 (Table 2). Therefore, the *Tgfbr1^*G*188V/+^* mouse was analyzed as an LDS model.

**TABLE 2:** Number of mice with each genotype.

**Genotype**	**Embryonic day 13.5**	**Embryonic day 14.5**	**Postnatal day 1**
Wildtype	12 (24%)	15 (29%)	63 (31%)
*Tgfbr1^*G*188V/+^*	25 (51%)	23 (45%)	139 (68.5%)
*Tgfbr1^*G*188V/G188V^*	12 (24%)	13 (26%)	1 (0.5%)*

*p < 0.001 vs. embryonic days 13.5 and 14.5.

### Pathophysiological Analysis of LDS Model Mice and Analysis of TGF-β Signaling in Response to the *Tgfbr1* G188V Mutation

**FIGURE 4:**
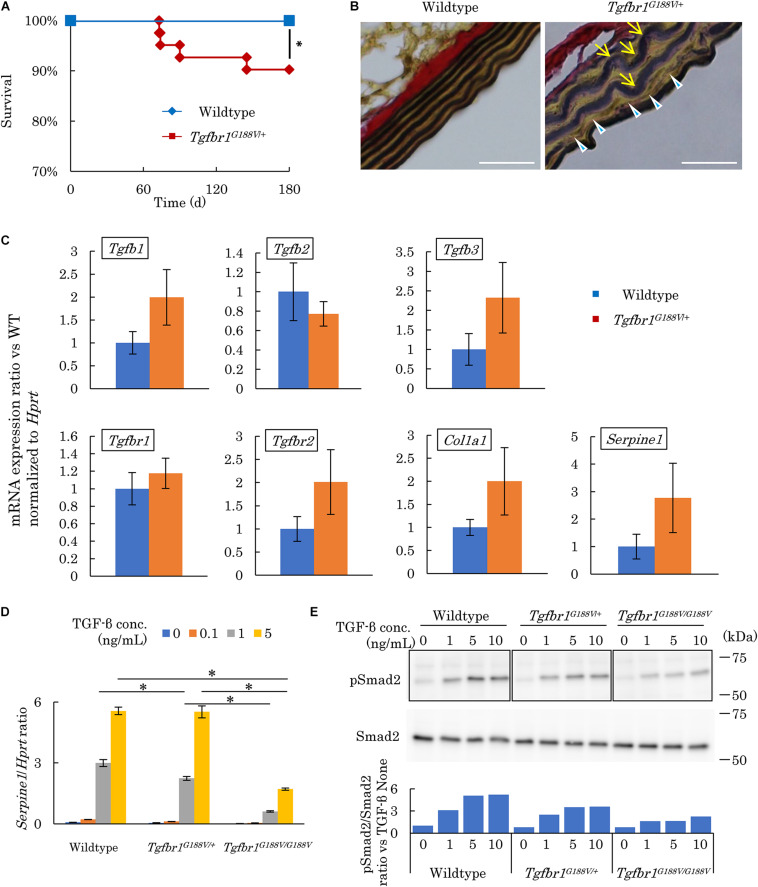
*Tgfbr1^*G*188V/+^* mice recapitulate vascular LDS phenotypes. **(A)** Kaplan–Meier survival curve showing a diminishing lifespan of *Tgfbr1^*G*188V/+^* mice. ^∗^*P* < 0.05. **(B)** Representative aortic wall sections of *Tgfbr1^*G*188V/+^* mice at 24 weeks of age stained with EVG to identify elastin fibers. Scale bar: 25 μm. Arrows show elastic fiber distortion. Arrowheads show elastic fiber fragmentation. **(C)** Analysis of TGF-β-related genes in aortic tissues derived from wildtype and *Tgfbr1^*G*188V/+^* mice (*n* = 4). Data represent means ± SD in triplicate assays. **(D)** *Serpine1* gene expression in MEFs derived from wildtype, *Tgfbr1^*G*188V/+^*, and *Tgfbr1^*G*188V/G188V^* embryos at embryonic day 13.5 stimulated with TGF-β (0–5 ng/mL). Data represent means ± SD in triplicate assays. ^∗^*P* < 0.05, compared with the indicated TGF-β concentration. **(E)** Phospho-Smad2 (pSmad2) in MEFs derived from wildtype, *Tgfbr1^*G*188V/+^*, and *Tgfbr1^*G*188V/G188V^* embryos at embryonic day 13.5 stimulated with TGF-β (0–10 ng/mL). Quantitative western blot analysis is shown as the ratios of the intensities of phospho-Smad2 to Smad2.

The survival rate of *Tgfbr1^*G*188V/+^* mice was observed over 180 days by a Kaplan–Meier survival curve. Mutant mice had died substantially earlier than WT mice (Figure 4A). To analyze the cause of premature death in *Tgfbr1^*G*188V/+^* mice, tissue sections of the aorta were prepared, and elastic fibers were observed. Aortae were collected from 24-week-old male WT and *Tgfbr1^*G*188V/+^* mice. Thinly sectioned axial specimens were prepared for EVG staining. In aortic samples from *Tgfbr1^*G*188V/+^* mice, elastic fibers appeared to be distorted (Figure 4B, arrow) and fine rupture of elastic fibers was observed (Figure 4B, arrowhead). Next, TGF-β-related gene expression was analyzed in aortae from *Tgfbr1^*G*188V/+^* mice. The thoracic aortae of 24-week-old male WT and *Tgfbr1^*G*188V/+^* mice were collected. mRNA expression levels of *Tgfb1*, *Tgfb2*, and *Tgfb3*, *TGF-*β receptor genes *Tgfbr1* and *Tgfbr2*, and TGF-β targets *Col1a1* and *Serpine1* were analyzed by real-time PCR. Although the differences were not statistically significant, the mRNA levels of *Tgfb1, Tgfb3, Tgfbr1, Tgfbr2*, *Col1a1*, and *Serpine1* tended to be higher in *Tgfbr1^*G*188V/+^* mice than in WT mice (Figure 4C). Using MEFs, we analyzed changes in TGF-β activity caused by the Tgfbr1 G188V mutation. MEFs of each genotype were cultured for 24 h in the absence of FBS and then stimulated with TGF-β (0–5 ng/mL). The expression levels of TGF-β-induced *Serpine1* were evaluated after 12 h (Figure 4D). In WT MEFs, *Serpine1* expression was increased in a TGF-β concentration-dependent manner. In *Tgfbr1^*G*188V/+^* and *Tgfbr1^*G*188V/G188V^* MEFs, the increase in *Serpine1* expression induced by TGF-β was reduced significantly. Next, we performed intracellular signal transduction analysis of TGF-β in MEFs. MEFs were cultured for 24 h in the absence of FBS and then stimulated with TGF-β (0–10 ng/mL). Cells were recovered after 30 min and Smad2 phosphorylation was examined by western blotting. Although phosphorylation of Smad2 in WT MEFs was increased in a TGF-β concentration-dependent manner, the levels of Smad2 phosphorylation were lower in *Tgfbr1^*G*188V/+^* and *Tgfbr1^*G*188V/G188V^* MEFs than in WT MEFs (Figure 4E). These findings demonstrated that cellular TGF-β responses had decreased because of the *Tgfbr1* G188V mutation.

### Analysis of Phenotypes in Periodontal Tissues of LDS Model Mice

**FIGURE 5:**
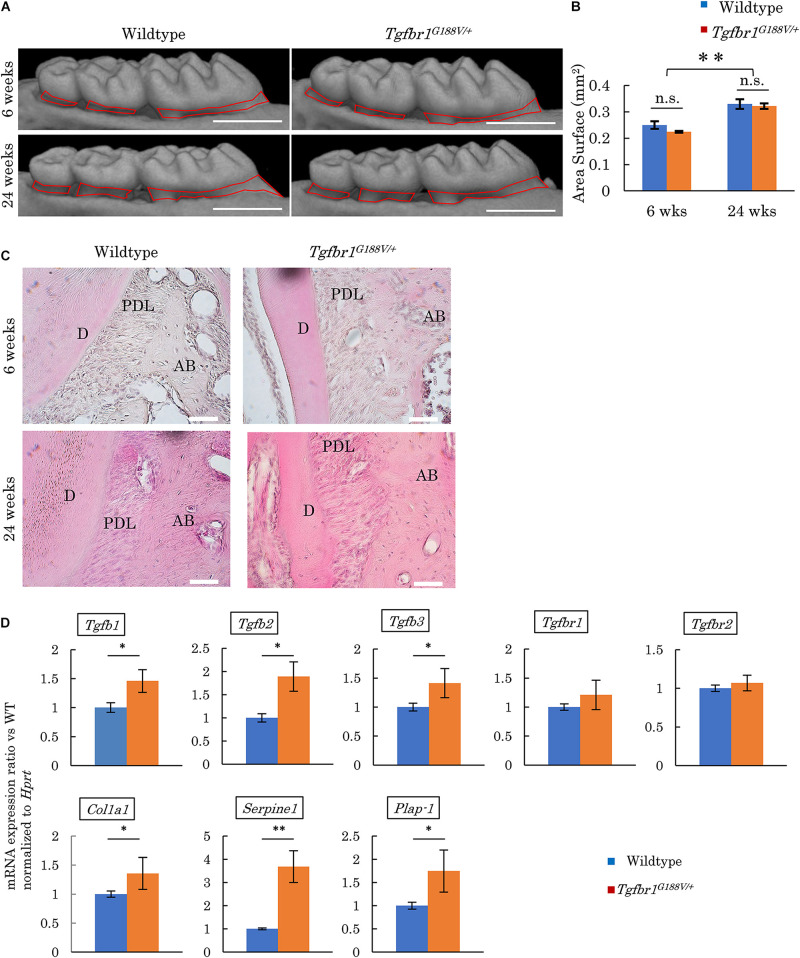
Analysis of phenotypes in periodontal tissues of *Tgfbr1^*G*188V/+^* mice. **(A)** Representative images from micro-CT analysis of maxillary alveolar bones in *Tgfbr1^*G*188V/+^* mice. Scale bar: 1 mm. **(B)** Alveolar bone resorption was measured in the root surface area between the alveolar apex from the cementoenamel junction (red line in Figure 4A). Data represent means ± SD. Six-week-old wildtype: *n* = 6, 6-week-old *Tgfbr1^*G*188V/+^*: *n* = 8, 24-week-old wildtype: *n* = 20, and 24-week-old *Tgfbr1^*G*188V/+^*: *n* = 14. **(C)** Representative images of HE staining of the periodontium from WT and *Tgfbr1^*G*188V/+^* mice at 6 and 24 weeks of age. AB, alveolar bone; PDL, periodontal ligament; D, dentin. Scale bar: 50 μm. **(D)** Analysis of TGF-β-related gene expression in periodontal tissues derived from wildtype and *Tgfbr1^*G*188V/+^* mice (*n* = 4). Data represent means ± SD in triplicate assays. ^∗^*P* < 0.05, ^∗∗^*P* < 0.01, compared with wildtype.

Micro-CT contrast imaging was performed to evaluate maxillary alveolar bones collected from WT and *Tgfbr1^*G*188V/+^* mice. Alveolar bone resorption was measured in the area between the alveolar apex from the cementoenamel boundary (Figure 5A, red line). The sums of values obtained for the first, second, and third molars were calculated. WT and *Tgfbr1^*G*188V/+^* mice showed significant increases in bone resorption with aging. However, there was no significant difference in alveolar bone resorption between WT and *Tgfbr1^*G*188V/+^* mice (Figure 5B). HE staining of periodontal tissues derived from WT and *Tgfbr1^*G*188V/+^* mice at 6 and 24 weeks of age revealed no structural differences between WT and *Tgfbr1^*G*188V/+^* mice (Figure 5C). Next, TGF-β-related gene expression was analyzed in periodontal tissues from *Tgfbr1^*G*188V/+^* mice. Periodontal tissues were collected from 6-week-old male WT and *Tgfbr1^*G*188V/+^* mice, and mRNA expression levels of TGF-β-related genes were analyzed by real-time PCR. Expression levels of *Tgfb1*, *Tgfb2*, *Tgfb3*, *Col1a1*, *Serpine*1, and *Plap-1* were significantly higher in *Tgfbr1^*G*188V/+^* mice than in WT mice (Figure 5D).

### Alveolar Bone Resorption in *Tgfbr1^*G*188V/+^* Mice in Response to Oral Infection

**FIGURE 6:**
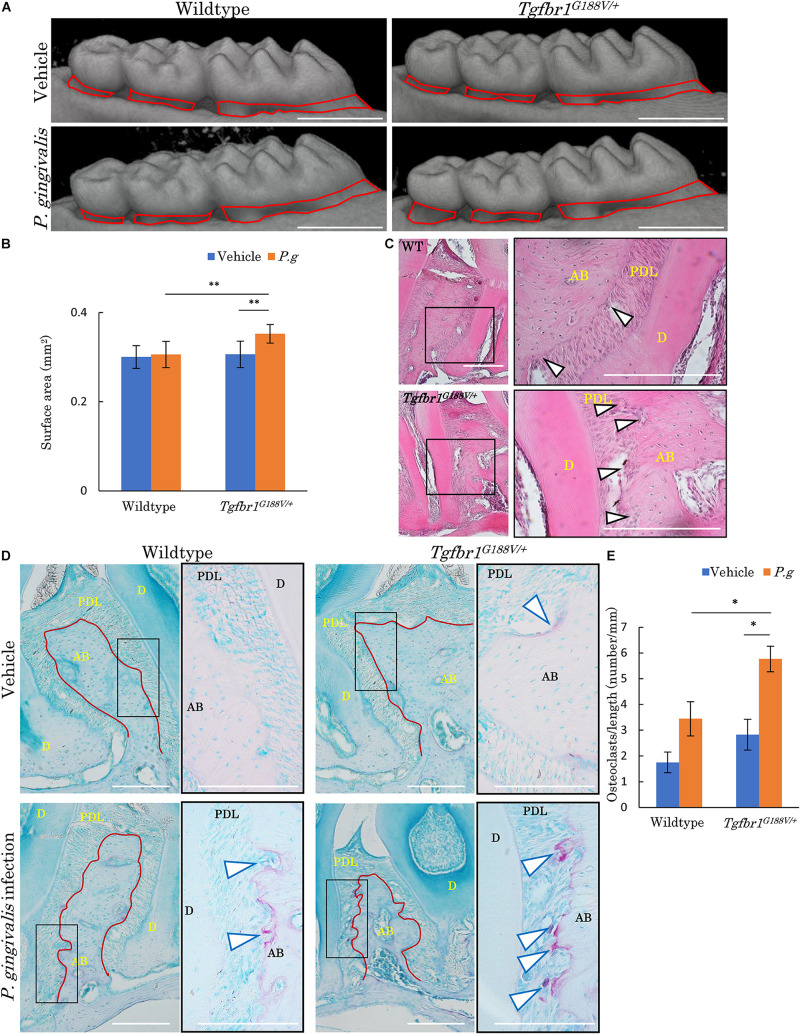
Oral infection with *P. gingivalis* induces alveolar bone resorption in *Tgfbr1^*G*188V/^*^+^ mice. **(A)** Representative images from micro-CT analysis of maxillary alveolar bones in *P. gingivalis*-infected WT and *Tgfbr1^*G*188V/+^* mice. Scale bar: 1 mm. **(B)** Alveolar bone resorption was measured in the root surface area between the alveolar apex from the cementoenamel junction (red line in **A**). Data represent means ± SD in triplicate assays. ^∗∗^*P* < 0.01. Wildtype: *n* = 11 in vehicle, *n* = 6 in *P. gingivalis* infection. **(C)** Representative images of HE staining of the periodontium from *P. gingivalis*-infected wildtype and *Tgfbr1^*G*188V/+^* mice. Right panel shows a high magnification images of the rectangular area indicated in the left panel. Scale bar: 200 μm. Arrowheads indicate multinucleated cells. AB, alveolar bone; PDL, periodontal ligament; D, dentin. **(D)** Representative images of TRAP-stained periodontium from *P. gingivalis*-infected wildtype and *Tgfbr1^*G*188V/+^* mice. Right panel shows a high magnification image of the rectangular area indicated in the left panel. Scale bar: 200 μm. Arrowheads indicate TRAP-positive multinucleated cells. **(E)** Quantification of TRAP-positive cell numbers in each group. TRAP-positive cell numbers on the surface of alveolar bone were counted and divided by the length of the alveolar bone (red line in **D**). Data represent means ± SD in wildtype: *n* = 3 in vehicle, *n* = 5 in *P. gingivalis* infection. *Tgfbr1^*G*188V/+^*: *n* = 4 in vehicle, *n* = 6 in *P. gingivalis* infection. Vehicle: sham control, *P.g*, *P. gingivalis* infection, AB, alveolar bone; D, dentin; PDL, periodontal ligament. ^∗^*P* < 0.05, compared with Vehicle.

To analyze changes in periodontal tissue caused by bacterial invasion in WT and *Tgfbr1^*G*188V/+^* mice, *P. gingivalis* was orally administered to generate an experimental periodontitis model. After *P. gingivalis* administration, the maxillary bones of the mice were recovered, micro-CT contrast imaging of periodontal tissues was performed (Figure 6A), and sections were prepared. Bone resorption after *P. gingivalis* administration was significantly greater in *Tgfbr1^*G*188V/+^* mice than in control mice (Figure 6B). Periodontal tissue sections were prepared and subjected to HE staining. Multinucleated cells near the alveolar bone were observed in WT and *Tgfbr1^*G*188V/+^* mice in the *P. gingivalis* treatment group (Figure 6C). To observe osteoclasts in close proximity to the alveolar bone, sections of each periodontal tissue were prepared, and TRAP staining was performed. In the *P. gingivalis* treatment group, osteoclasts were found near the alveolar bone (Figure 6D, arrowhead). For quantitative analysis, the numbers of osteoclasts in close proximity to the alveolar bone were counted between the first and second molars and between the second and third molars. These values were divided by the length of the alveolar bone surface for comparison (Figure 6D, red line). In *Tgfbr1^*G*188V/+^* mice, the number of osteoclasts was increased significantly after *P. gingivalis* administration (Figure 6E).

### Upregulation of Inflammatory Cytokine mRNA Expression in Peritoneal Macrophages From *Tgfbr1^*G*188V/+^* Mice

**FIGURE 7:**
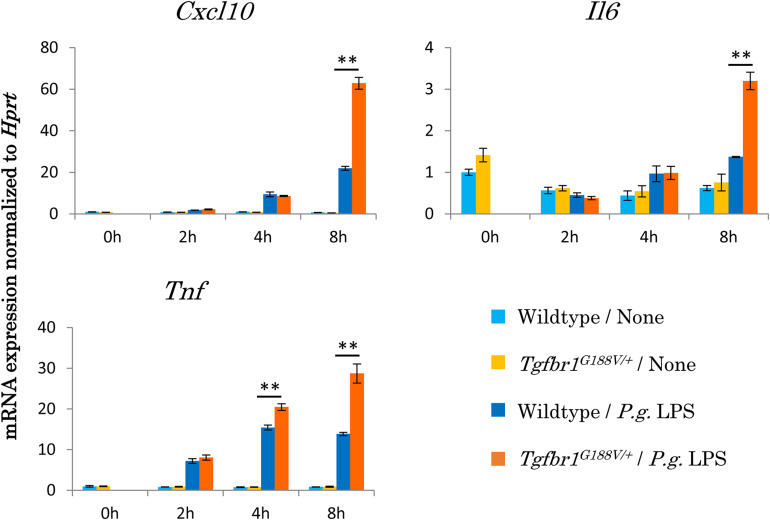
Upregulation of inflammatory cytokine mRNA expression in peritoneal macrophages from *Tgfbr1^*G*188V/+^* mice. Macrophages were harvested from the peritoneal cavity of 8-week-old male WT and *Tgfbr1^*G*188V/+^* mice at 3 days after injection of thioglycolate. The peritoneal macrophages were stimulated with 1 μg/ml *P. gingivalis* LPS for the indicated times and then total RNA was isolated for quantitative PCR analysis. Data represent means ± SD in triplicate assays. ^∗∗^*P* < 0.01, compared with wildtype.

To assess the innate immunological response of *Tgfbr1^*G*188V/+^* mice to *P. gingivalis*, we isolated peritoneal macrophages from WT and *Tgfbr1^*G*188V/+^* mice, stimulated these cells with *P. gingivalis* LPS, and then analyzed inflammatory cytokine expression by quantitative PCR (Figure 7). We found that mRNA expression of *Cxcl10*, *Il6* and *Tnf* was significantly upregulated in *Tgfbr1^*G*188V/+^* macrophages compared with WT macrophages.

## Discussion

In this study, we generated a knock-in mouse that reproduced the *TGFBR1* gene mutation in an LDS patient who exhibited localized and remarkable vertical bone resorption, despite generally good plaque control. We then analyzed the phenotypes of this mouse model. Aggressive periodontitis is strongly affected by genetic factors and the morbidity rate in Japan is 0.05–1% (Takahashi et al., 2011). More than 40 genetic variants associated with LDS have been reported thus far (Loeys et al., 2006). However, the patient in this study had a previously unreported mutation, namely a novel G188V mutation in the GS domain of *TGFBR1*. TGF-β binds to type 2 receptors on cell membranes, thereby forming a complex with type 1 receptors. Type 2 receptors have serine–threonine kinase activity in the intracellular region and phosphorylate the GS domain of bound type 1 receptors. The GS domain of type 1 receptors is phosphorylated and kinase activation results in signal transmission to cells via R-Smad, an intracellular signal transduction molecule (Doyle et al., 2012). The genetic mutation identified in the LDS patient in this study attenuated Smad signaling. Structural changes in the GS domain of TGFBR1 may prevent phosphorylation of the GS domain and inhibit signal transduction. However, distinct point mutations in *TGFBR1*, which spontaneously activate TGF-β signaling, have been reported as the cause of multiple self-healing squamous epithelioma, also known as Ferguson–Smith disease (Goudie et al., 2011). The detailed changes in receptor locality and the three-dimensional structure are unclear and should be examined in future studies.

In the mouse model established in this study, the *Tgfbr1* G188V/G188V mutation was lethal in embryos. Similarly, this mutation has not been identified in human homozygous patients with LDS. Therefore, Tgfbr1 is considered to be essential for survival. Some *Tgfbr1^*G*188V/G188V^* embryos collected at day 14.5 showed fewer bloodstreams in craniofacial, trunk, and spinal cord regions. It is important to investigate the effects of the *Tgfbr1* G188V/G188V mutation on cardiovascular development during embryogenesis to reveal the mechanisms of embryonic lethality. In *Tgfbr1^*G*188V/+^* mice, distortion and fine tearing of elastic fibers in aortic tissue were confirmed and a tendency toward premature death was observed. Structural abnormalities of the elastic fibers of the aorta trigger pathological diseases (e.g., aortic aneurysm, aortic dissection, hemorrhage, and fibrosis) and premature death. However, because the relationship between aortic structural abnormalities and aortic aneurysm was not examined, additional studies are necessary.

In this study, *Tgfbr1^*G*188V/+^* MEFs showed attenuation of TGF-β signaling, but had elevated TGF-β-related gene expression in aortic tissues. In studies of aortic tissues, overexpression of TGF-β has been reported in patients with LDS and an LDS mouse model (Loeys et al., 2005, 2006; Gallo et al., 2014). TGF-β signaling involves canonical pathways via Smad and non-canonical pathways via ERK, JNK, and other molecules. Their expression patterns are regulated by a feedback mechanism that involves Smad (Lindsay and Dietz, 2011). In LDS, mutations in TGF-β receptors selectively attenuate the canonical pathway, thereby inhibiting the negative feedback mechanism that underlies TGF-β expression. Accordingly, increased levels of TGF-β in tissues contribute to excessive canonical pathway signaling. Non-canonical pathways are also presumed to cause aortic lesions by excessive activation (Lindsay and Dietz, 2011). In *Tgfbr1^*G*188V/+^* mice, aortic elastic fiber abnormalities likely occurred through the same mechanism.

Periodontal tissues showed significantly higher expression levels of TGF-β-related genes in *Tgfbr1^*G*188V/+^* mice than in WT mice, with no obvious differences in alveolar bone resorption or periodontal tissue status. TGF-β overexpression in tissues was presumed to compensate for the decrease in TGF-β signaling at the cellular level similar to the findings in aortic tissues. However, no apparent periodontal tissue-specific structural abnormalities were observed by HE staining. Therefore, abnormalities related to excessive TGF-β signaling were not triggered in periodontal tissues without pathological stimulation. In the future, detailed analyses at cellular and molecular levels are needed to evaluate structural abnormalities in periodontal tissues.

In the *P. gingivalis*-induced periodontitis model, significantly more osteoclasts were present in *Tgfbr1^*G*188V/+^* mice than in WT mice. Moreover, alveolar bone resorption was significantly increased in *Tgfbr1^*G*188V/+^* mice. *P. gingivalis* is commonly used to induce periodontitis (Baker et al., 1994; Suda et al., 2013; Arimatsu et al., 2014; Papathanasiou et al., 2016). In this experimental periodontitis model, the oral administration of *P. gingivalis* causes changes in the intestinal flora, which leads to systemic inflammation and subsequent bone resorption through immune cell responses in the oral cavity (Baker et al., 1994; Arimatsu et al., 2014). Notably, the oral administration of *P. gingivalis* may have triggered systemic inflammation in *Tgfbr1^*G*188V/+^* mice. A previous report has demonstrated that TGF-β signaling in macrophages suppresses Toll-like receptor (TLR) signaling through myeloid differentiation factor 88 (Naiki et al., 2005). In *Tgfbr1^*G*188V/+^* mouse macrophages, the mutation in *Tgfbr1* may promote TLR signaling by attenuation of TGF-β signaling, thereby increasing the macrophage response to *P. gingivalis* LPS intrinsically. In the future, more details of the mechanisms that underlie osteoclastogenesis induced by *P. gingivalis* in *Tgfbr1^*G*188V/+^* mice should be analyzed to understand the pathophysiology of periodontal disease caused by the *Tgfbr1^*G*188V/+^* mutation.

Smad-mediated TGF-β signaling inhibits osteoblast differentiation (Borton et al., 2001). In periodontal tissues of *Tgfbr1^*G*188V/+^* mice, excessive TGF-β signaling may suppress osteoblast differentiation. In LDS model mice with mutations in TGF-β II receptors, the femur is thinner and bone mass decreases, thereby increasing fragility (Dewan et al., 2015). In *Tgfbr1^*G*188V/+^* mice, cell differentiation into osteoblasts and osteocytes may be suppressed, which suggests that the alveolar bone tends to be susceptible to resorption.

## Conclusion

In conclusion we established an LDS mouse model that showed elevated susceptibility to *P. gingivalis*-induced periodontitis, probably through TGF-β signal dysfunction. This suggests that TGF-β signaling abnormalities accelerate the pathogenesis or progression of periodontitis.

## Data Availability Statement

The original contributions presented in the study are included in the article/supplementary material, further inquiries can be directed to the corresponding author/s.

## Ethics Statement

The studies involving human participants were reviewed and approved by the Institutional Ethics Committee of Osaka University Graduate School of Dentistry, and National Cerebral and Cardiovascular Center. The patients/participants provided their written informed consent to participate in this study. The animal study was reviewed and approved by Institutional Animal Care and Use Committee of Osaka University Graduate School of Dentistry.

## Author Contributions

SY, KT, TM, and SM conceived, designed the experiments, and wrote the manuscript. SY, KT, MK, HS, and TK performed the experiments. SY, KT, CF, TM, HY, SS, and SM analyzed the data. All authors contributed to the article and approved the submitted version.

## Conflict of Interest

The authors declare that the research was conducted in the absence of any commercial or financial relationships that could be construed as a potential conflict of interest.

## Publisher’s Note

All claims expressed in this article are solely those of the authors and do not necessarily represent those of their affiliated organizations, or those of the publisher, the editors and the reviewers. Any product that may be evaluated in this article, or claim that may be made by its manufacturer, is not guaranteed or endorsed by the publisher.
